# Defined Composition of Culture Media Promotes Rodent Neonatal Cardiomyocyte Maturation and Enables Functional Neuro-Cardiac Co-Culture

**DOI:** 10.3390/cells14181434

**Published:** 2025-09-13

**Authors:** Giulia Borile, Lolita Dokshokova, Nicola Moro, Antonio Campo, Valentina Prando, Jose L. Sanchez-Alonso, Julia Gorelik, Giuseppe Faggian, Marco Mongillo, Tania Zaglia

**Affiliations:** 1 Department of Biomedical Sciences, University of Padova, 35131 Padova, Italy; 2 Veneto Institute of Molecular Medicine, 35129 Padova, Italy; 3 National Heart and Lung Institute, London SW3 6LY, UK; 4 Division of Cardiac Surgery, University of Verona, 37126 Verona, Italy

**Keywords:** neonatal cardiomyocytes, in vitro maturation, sympathetic neurons, neuro-cardiac junctions, co-culture model, calcium imaging

## Abstract

Neonatal rodent cardiomyocytes (CMs) are a mainstay of in vitro cardiac research, yet their immature phenotype limits the study of key physiological processes such as excitation–contraction coupling (ECC) and sympathetic modulation. Here, we present a defined low-glucose, serum-free (LGSF) culture protocol that drives the structural and functional maturation of neonatal CMs and supports their integration into functional neuro-cardiac co-cultures. After 15 days in LGSF conditions, CMs exhibit elongated morphology, organized sarcomeres, polarized connexin-43, mitochondrial redistribution, and sarcoplasmic reticulum (SR) development, all closely resembling features of adult cells. These structural hallmarks were paralleled by enhanced Ca^2^^+^ handling, with increased SR contribution and reduced spontaneous activity, indicative of a mature ECC phenotype. When co-cultured with sympathetic neurons (SN), CMs established anatomically distinct neuro-cardiac junctions. Notably, nicotine stimulation triggered spatially restricted, reversible increases in CM Ca^2^^+^ transients, confined to varicosity-contacted cells. Pharmacological analysis revealed subtype-specific roles for β_1_- and β_2_-adrenergic receptors, and uncovered evidence of functional crosstalk between them. Our study defines a reproducible culture framework that advances CM maturation and enables the high-resolution interrogation of synaptic-like sympathetic modulation. This approach opens new avenues for mechanistic studies and drug testing in developmentally relevant neuro-cardiac systems.

## 1. Introduction

Cardiomyocytes (CMs) are highly specialized cells whose morphology, contractile properties, and signaling dynamics are profoundly influenced by their microenvironment. Various in vitro CM models have proven indispensable for studying cardiac physiology and pathology, yet faithfully preserving native cell features remains challenging, as each model involves inherent trade-offs. Adult CMs remain the gold standard due to their native architecture and mature electrophysiological properties, but they are notoriously fragile, technically demanding to isolate, and unsuitable for long-term studies [1]. Human-induced pluripotent stem cell-derived cardiomyocytes (hiPSC-CM) provide an unlimited, patient-specific source and can be specified into ventricular, atrial, or nodal subtypes [2]. However, they retain a fetal-like phenotype with disorganized sarcomeres, immature T-tubules and sarcoplasmic reticulum (SR), suboptimal Ca^2^^+^ handling, and slow maturation [3]. Neonatal rodent CMs, first described by Harary and Farley [4], offer a pragmatic compromise. They are easily obtainable, genetically and pharmacologically accessible, and stable in culture for weeks, features that make them ideal for rapid, mechanistic studies. Their structural and functional immaturity compared to adult CMs is a known limitation, but targeted culture optimization can partially enhance their maturation. Beyond being a convenient CM model, neonatal rodent CMs have played a pivotal role in exploring neuro-cardiac communication, where the dialogue between CMs and sympathetic neurons (SNs) shapes both physiological and pathological cardiac responses [5,6,7]. The ventricular myocardium is densely innervated by SNs, which form intricate networks with varicosities, specialized contact sites referred to as neuro-cardiac junctions (NCJs) [8,9,10,11]. NCJs are not static anatomical structures; they are dynamic hubs for bidirectional communication. SNs modulate CMs’ excitability, contractility, and trophism (anterograde signaling), while CMs influence neuronal growth and function (retrograde signaling) [8,9,10,11]. Alterations in sympathetic innervation are frequently associated with—and may contribute to—the mechanisms of cardiac disease, including myocardial infarction, hypertrophy, and arrhythmias [12,13,14,15]. Such remodeling of sympathetic fibers contributes to electrophysiological instability, arrhythmogenicity, and disease progression. Understanding how NCJs form and function is therefore essential for identifying novel therapeutic targets. While co-cultures of hiPSC-derived cardiomyocytes and SNs can be established, they generally require prolonged, resource-intensive protocols to achieve mutual maturation [16,17,18,19]. In contrast, neonatal CM-based systems offer greater speed and scalability, but often lack the structural maturity needed to faithfully model NCJ-driven Ca^2^^+^ dynamics. During the development of SN–CM co-cultures [9,10,11], we observed that culturing cells in a low-glucose, serum-free (LGSF) medium significantly improved CM morphology, Ca^2^^+^ dynamics, and overall performance, while supporting stable and functional neuro-cardiac connectivity. Therefore, here we systematically evaluate the formulation, rationale, and impact of a culture medium designed to enhance rodent neonatal CM maturation through metabolic restriction (low glucose) and serum deprivation, aiming to limit non-myocyte overgrowth and create a more physiological platform for neuro-cardiac studies. We assess structural organization, organelle distribution, and excitation–contraction coupling using confocal imaging, molecular profiling, and live-cell Ca^2^^+^ recordings. We further evaluate whether NCJs formed under defined conditions mediate localized, neuron-triggered Ca^2^^+^ responses.

Our findings establish a robust and scalable co-culture system that faithfully captures key aspects of SN–CM communication under controlled conditions, bridging a critical gap between reductionist models and the complexity of in vivo innervation.

## 2. Materials and Methods

### 2.1. Ethical Approval

All experimental procedures involving animals were approved by the Italian Ministry of Health (Ufficio VI) in accordance with national and European legislation on animal welfare (approval number A06E0.N.JJ6). All experiments were performed by trained personnel with certified experience in laboratory animal handling. Procedures were refined to minimize animal discomfort, and sample sizes were determined based on power analysis to ensure statistical significance while reducing animal usage.

### 2.2. Origin of Animals

P1–P3 Sprague Dawley rats (Charles River, Milan, Italy) were used in this study. Animals were housed in individually ventilated cages within an authorized animal facility (authorization number 175/2002A), under a controlled 12:12 h light/dark cycle, with ad libitum access to food and water. Neonatal rats were euthanized via cervical dislocation in accordance with Annex IV of Directive 2010/63/EU.

### 2.3. Isolation and Culture of Newborn Rat Ventricular Cardiomyocytes

Ventricular CMs were isolated from P1–P3 rats as previously described [9,10,11,20]. Following euthanasia, hearts were excised, atria removed, and ventricles transferred to ice-cold ADS buffer (in mM: 106 NaCl, 20 HEPES, 0.8 Na_2_HPO_4_, 5.3 KCl, 0.4 MgSO_4_, 5 D-glucose; all from Sigma Aldrich, St. Louis, MO, USA). Tissues were minced and subjected to enzymatic digestion in six sequential 20 min cycles at 37 °C using an enzymatic solution containing 0.45 mg/mL collagenase A and 1.25 mg/mL pancreatin (Sigma Aldrich, St. Louis, MO, USA). After each cycle, the supernatant was collected and enzyme activity quenched with 1.5 mL horse serum. The pooled cell suspensions were centrifuged at 330× *g* (slow brake), resuspended in first-day culture medium, and pre-plated for 1 h at 37 °C to reduce fibroblast contamination. Non-adherent cells were collected, counted, and plated on mouse laminin-coated surfaces (1.8 mg/100 mm^2^; Corning, NY, USA) at 470 cells/mm^2^ for imaging and 550 cells/mm^2^ for biochemical assays. After 24 h, unattached cells were removed by washing with warm ADS, and media were replaced with second-day formulations. To inhibit fibroblast proliferation, 100 µM 5-bromo-2′-deoxyuridine (BrdU; Sigma Aldrich, St. Louis, MO, USA) was added during the first two days. Media were refreshed every 48 h. To enhance CMs’ structural and functional maturation, a defined low-glucose, serum-free (LGSF) medium was developed in-house to replace conventional high-glucose, serum-rich conditions (see Table 1 and Results Section 3.1).

### 2.4. Establishment of SN/CM Co-Cultures

SNs were isolated from the superior cervical ganglia (SCG) of P1–P3 Sprague Dawley rats following a modified version of the protocol by Zareen and Greene [21]. After heart removal, SCGs were carefully dissected and transferred to ice-cold complete medium (MEM 85%, HS 10%, FBS 5%; all from Thermo Fisher Scientific, Waltham, MA, USA). Ganglia were collected in a 15 mL tube, allowed to settle using gravity, and the supernatant was discarded. Tissues were then incubated in 0.25% *w*/*v* trypsin-EDTA (Thermo Fisher Scientific, Waltham, MA, USA) for 30 min at 37 °C. Enzymatic digestion was stopped by adding 10 mL of complete medium. Ganglia were centrifuged at 100× *g* for 2 min, resuspended in 1 mL of first-day LGSF medium, and dissociated using fire-polished glass pipettes of decreasing bore size. Undigested tissue fragments were allowed to settle, and the supernatant containing dissociated neurons was collected and cells counted. Neurons were mixed with neonatal CMs at a 1:50 SN:CM ratio and plated on laminin-coated surfaces (1.8 mg/100 mm^2^; Corning) at a final density of 480 cells/mm^2^ in LGSF medium supplemented with 100 ng/mL nerve growth factor (NGF-β; Sigma Aldrich, St. Louis, MO, USA). After 24 h, the medium was replaced with second-day LGSF medium containing NGF (100 ng/mL). From day 2 onward, half of the medium was refreshed every 48 h. CMs were classified as innervated when in direct physical contact with Tyrosine Hydroxylase (TH)-positive sympathetic fibers, as determined by confocal immunofluorescence (α-actinin-positive cardiomyocytes, TH-positive neurons). This morphological classification was validated functionally, since only innervated cells exhibited Ca^2^^+^ responses to nicotinic stimulation [9].

### 2.5. Immunofluorescence in Cultured Cells

Cells were fixed in 4% (*w*/*v*) paraformaldehyde (PFA; Sigma Aldrich, St. Louis, MO, USA) in phosphate-buffered saline (PBS) for 30 min at room temperature (RT). Permeabilization was performed in PBS containing 1% (*w*/*v*) bovine serum albumin (BSA; Sigma Aldrich, St. Louis, MO, USA) and 0.1% (*v*/*v*) Triton X-100 for 5 min. Cells were incubated with primary antibodies diluted in PBS with 1% BSA for 2 h at 37 °C in a humidified chamber. After washing, appropriate fluorescent-conjugated secondary antibodies were applied for 30 min at 37 °C. Nuclei were counterstained with 4′,6-diamidino-2-phenylindole (DAPI, 100 ng/mL in PBS; Sigma Aldrich, St. Louis, MO, USA) for 5 min at RT. Imaging was performed using confocal microscopes (Leica TCS SP5 or Zeiss LSM900; Leica Microsystems, Wetzlar, Germany; Carl Zeiss, Oberkochen, Germany). Antibody details are listed in Table 2.

### 2.6. Morphometric Analyses

Confocal images of neonatal CMs cultured in standard or LGSF media were analyzed using ImageJ (v1.54 NIH, Bethesda, MD, USA). The following morphological parameters were quantified: (i) cell area, (ii) aspect ratio, (iii) roundness, (iv) solidity, and (v) sarcomeric organization, the latter based on fluorescence intensity plot profiles. For each condition, twelve randomly selected fields were acquired and analyzed per experiment. Morphometric analyses were performed on at least three independent biological replicates.

### 2.7. Transmission Electron Microscopy

Cells were fixed in 2.5% (*v*/*v*) glutaraldehyde in 0.1 M sodium cacodylate buffer for 1 h at 4 °C, then washed twice (30 min each) in 0.2 M sucrose and 0.1 M sodium cacodylate (all reagents from Sigma Aldrich, St. Louis, MO, USA). Post-fixation was performed in 1% (*w*/*v*) osmium tetroxide in 0.1 M sodium cacodylate for 2 h at 4 °C (all reagents from Sigma Aldrich, St. Louis, MO, USA). Samples were dehydrated through graded ethanol series, treated with propylene oxide for 45 min, and embedded in epoxy resin. Semi-thin sections were obtained, stained with uranyl acetate (50% ethanol) and Reynolds lead citrate, and analyzed using a FEI Tecnai 12 transmission electron microscope.

### 2.8. Real-Time Quantitative PCR

Total RNA was extracted using the SV Total RNA Isolation System (Promega, Madison, WI, USA), following the manufacturer’s protocol. First-strand cDNA synthesis was performed with SuperScript III Reverse Transcriptase (Thermo Fisher Scientific, Waltham, MA, USA). RT-qPCR was conducted using a QuantStudio 5 Real-Time PCR System (Thermo Fisher Scientific, Waltham, MA, USA). Gene-specific primers are listed in Table 3. Data were analyzed using the ΔΔCt method, and results were normalized to Gapdh expression. PCR cycling conditions and analytical workflow were as previously described [22].

### 2.9. Western Blot in Neonatal CMs

Neonatal CMs were lysed in RIPA buffer (Sigma Aldrich, St. Louis, MO, USA) supplemented with protease and phosphatase inhibitors (cOmplete™ and PhosSTOP™, Sigma Aldrich, St. Louis, MO, USA). Lysates were incubated on ice for 1 h and centrifuged at 17,000× *g* for 15 min. Supernatants were collected and stored at −80 °C. Proteins were separated on 4–12% SDS-PAGE gels (Thermo Fisher Scientific, Waltham, MA, USA) and transferred to PVDF membranes (Amersham™ Hybond^®®^ P, Sigma Aldrich, St. Louis, MO, USA). Membranes were blocked in 5% (*w*/*v*) non-fat dry milk in Tris-Buffered Saline (TBS) with 0.1% Tween-20 (Sigma Aldrich, St. Louis, MO, USA) for 1 h at RT. Primary antibodies were applied overnight at 4 °C in blocking solution. HRP-conjugated secondary antibodies (BioRad, Hercules, CA, USA) were incubated for 90 min at RT. Signal detection was performed using Pierce™ ECL Plus substrate (Thermo Fisher Scientific, Waltham, MA, USA) and acquired using an ImageQuant™ LAS 4000 system (GE Healthcare, Chicago, IL, USA). Band intensities were quantified with ImageJ (v1.54 NIH, Bethesda, MD, USA).

### 2.10. Real-Time Ca^2+^ Imaging in Neonatal CM

Neonatal CMs were loaded with 2.5 µM Fluo-4 AM (Thermo Fisher Scientific, Waltham, MA, USA) in imaging solution containing (in mM): 125 NaCl, 20 HEPES, 1 Na_3_PO_4_, 5 KCl, 1 MgSO_4_, 5.5 D-glucose, and 1.8 CaCl_2_ (all from Sigma Aldrich, St. Louis, MO, USA). Cells were incubated for 20 min at 37 °C, washed with dye-free solution, and allowed to de-esterify for 10 min at 37 °C. Sulfinpyrazone (0.1 mM, Sigma Aldrich, St. Louis, MO, USA) was added throughout to prevent dye extrusion. For mitochondrial Ca^2+^ imaging, neonatal CMs were infected with an adenoviral vector coding for mito-GCaMP6f (courtesy of Prof. De Stefani) for 24 h, two days before the experiment. Coverslips were mounted in an RC-21BRFS chamber (Warner Instruments, Holliston, MA, USA) and imaged using a Leica TCS SP5 confocal microscope (Leica Microsystems, Wetzlar, Germany) equipped with a 63× oil-immersion objective (NA 1.4). CMs were field-stimulated at 1 Hz for 1 min to stabilize electrical activity, and spontaneous Ca^2^^+^ sparks were recorded immediately after pacing ceased. Fluo-4 was excited at 488 nm (argon laser) and collected at 525/40 nm using line-scan mode (400 Hz). Spark amplitude, full-width at half-maximum (FWHM), and full-duration at half-maximum (FDHM) were quantified using the SparkMaster [23] plugin in ImageJ (v1.54 NIH, Bethesda, MD, USA). For cytosolic or mitochondrial Ca^2+^ transients analyses, an inverted Olympus IX81 fluorescence microscope equipped with a 40x oil-immersion objective was used. To stimulate SR Ca^2+^ depletion, cells were superfused with imaging solution supplemented with 10 mM caffeine (Sigma Aldrich, St. Louis, MO, USA). Offline analyses were performed with ImageJ (v1.54 NIH, Bethesda, MD, USA) and Clampfit (v11.2, Molecular Devices, LLC., San Jose, CA, USA).

### 2.11. Real-Time Ca^2+^ Imaging in SN/CM Co-Cultures

SN/CM co-cultures were loaded with 1.5 µM Fluo-4 AM in Hank’s Balanced Salt Solution (HBSS; Thermo Fisher Scientific, Waltham, MA, USA), supplemented with 10 mM HEPES and 4 mM NaHCO_3_ (Sigma Aldrich, St. Louis, MO, USA), for 20 min at 37 °C. Following washing, cells were incubated in dye-free buffer for 10 min at 37 °C. SP (0.1 mM, Sigma Aldrich, St. Louis, MO, USA) was included throughout to limit dye leakage. Imaging was performed on an inverted fluorescence microscope (Nikon Instruments, Amstelveen, Netherlands) equipped with a 63× oil-immersion objective and a MICAM Ultima digital camera (SciMedia, Costa Mesa, CA, USA). Cells were stimulated at 0.5 Hz (30 V) to establish baseline Ca^2^^+^ transients. Neuronal activation was triggered by 1 µM nicotine (Tocris Bioscience, Bristol, UK), and Ca^2^^+^ responses were analyzed using BV Analyse v11.08 software (SciMedia, Costa Mesa, CA, USA). In selected experiments, β-adrenergic receptor (AR) antagonists were added: 50 nM ICI-118551 for β_2_AR and 300 nM CGP-20712 for β_1_AR (both from Sigma Aldrich, St. Louis, MO, USA).

### 2.12. Combined Live Imaging Ca^2+^ Dynamics and Scanning Ion Conductance Microscopy (SICM) in CM-SN Co-Cultures

SN/CM co-cultures were imaged using SICM combined with Fluo-4-based Ca^2^^+^ imaging, as previously described [24,25]. SICM was performed with an electrolyte-filled nanopipette scanning the cell surface, detecting changes in ion current proportional to pipette–membrane distance. Local stimulation was achieved by applying brief pressure pulses through the pipette to deliver ~1 µM nicotine (Tocris Bioscience, Bristol, UK) to individual SN soma. The stimulation buffer contained the following (in mM): 145 KCl, 2 MgCl_2_, and 5 HEPES (pH 7.4, all from Sigma Aldrich, St. Louis, MO, USA). During acquisition, cells were superfused with external solution composed of 145 KCl, 1 MgCl_2_, 1 CaCl_2_, 2 EGTA, 10 glucose, and 10 HEPES (pH 7.4, all from Sigma Aldrich, St. Louis, MO, USA). After nicotine application, Ca^2^^+^ transients were recorded in Fluo-4–loaded CM paced at 0.5 Hz and analyzed with BV Analyse v11.08 (SciMedia, Costa Mesa, CA, USA).

### 2.13. Statistical Analysis

Statistical analysis was performed using GraphPad Prism 8 (GraphPad Software, San Diego, CA, USA). Normality was assessed using the Shapiro–Wilk test. For comparison between two groups, an unpaired two-tailed Student’s *t*-test was applied if data were normally distributed with equal variances; Welch’s correction was used otherwise. Non-parametric data were analyzed using the Mann–Whitney test. For comparisons among ≥3 groups, one-way ANOVA with appropriate post hoc tests was used. Results are presented as mean ± standard deviation (SD) unless otherwise specified. A *p*-value ≤ 0.05 was considered statistically significant.

## 3. Results

### 3.1. Low-Glucose, Serum-Deprived Culture Medium Drives Structural and Functional Maturation of Rat Neonatal Cardiomyocytes

To promote the in vitro maturation of neonatal cardiomyocytes (CM) toward a more adult-like phenotype, we developed a low-glucose, serum-free (LGSF) medium in which glucose concentration was reduced and serum was replaced by specific supplements (insulin, transferrin, selenium). The isolation protocol remained unchanged (see Section 2.3). Serum removal, known to promote fibroblast proliferation, significantly increased culture purity, with 85 ± 5% of cells expressing α-actinin compared to 60 ± 5% under standard conditions, as quantified by the ratio of α-actinin–positive cells to total nuclei (Supplementary Figure S1A). This enrichment was further confirmed by Western blot analysis, which revealed increased α-actinin protein levels under LGSF conditions when equal amounts of total protein were loaded (Supplementary Figure S1B,C). We next assessed whether these defined conditions influenced CMs’ morphological and structural maturation. Higher CM purity also improved culture stability, enabling monolayers to be maintained for at least two weeks—and up to three weeks in selected preparations—thus supporting extended maturation protocols and long-term experimental applications. To preserve physiological stability over time, 50% of the medium was replaced every two days with a mix of fresh and pre-conditioned medium, which was cleared of macroscopic debris by centrifugation while retaining endogenous soluble factors.

#### 3.1.1. LGSF Medium Improves Cell Geometry and Sarcomeric Organization

**Figure 1:**
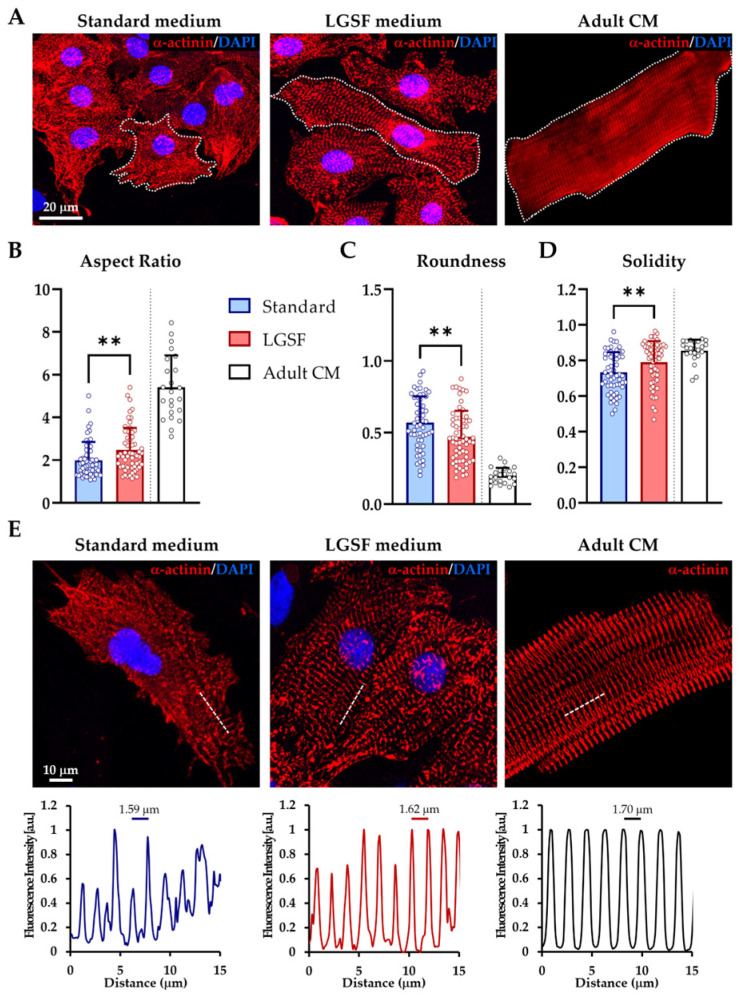
LGSF medium enhances the morphological maturation of neonatal rat cardiomyocytes (CMs). (**A**) Confocal images of CMs cultured for 15 days in standard medium (left) or low-glucose serum-free (LGSF) medium (middle), compared to freshly isolated adult cardiomyocytes (CMs, right). Cells were stained for α-actinin (red); nuclei were counterstained with 4′,6-diamidino-2-phenylindole (DAPI, blue). Dashed lines indicate individual cell borders. (**B**–**D**) Morphometric analysis of CM shape descriptors under standard (blue), LGSF (red), and adult (white) conditions. Data were analyzed by Mann–Whitney test (**, *p* ≤ 0.01; *n* > 50 cells/group). (**E**) Representative α-actinin staining with corresponding fluorescence intensity profiles (expressed as arbitrary units, a.u.) showing improved sarcomeric organization in LGSF and adult CMs. Data were collected from three independent culture experiments.

CM morphology was assessed by α-actinin immunostaining combined with a quantitative analysis of shape descriptors, including aspect ratio (elongation), roundness (deviation from circularity), and solidity (compactness, inversely related to membrane protrusions). CMs cultured in the standard medium exhibited a polygonal shape with multiple extensions, whereas cells maintained in the LGSF medium displayed an elongated, rod-like profile, more closely resembling adult CM morphology (Figure 1A–D). Cell area was also significantly increased in LGSF conditions (*p* ≤ 0.001; Table 4). Notably, aspect ratio and solidity were significantly higher in LGSF-cultured CMs, and approached the values measured in freshly isolated adult CMs, while roundness was correspondingly reduced (Table 4). These trends indicate a shift toward a more mature, adult-like geometry. To assess sarcomere organization, we analyzed α-actinin fluorescence intensity profiles across the cell. Fluorescence imaging demonstrated that CMs cultured in the standard medium displayed a heterogeneous sarcomeric pattern, with organized regions alternating with poorly defined ones. In contrast, CMs maintained in LGSF medium exhibited a more uniform and continuous arrangement of Z-bands, closely resembling that of adult cardiomyocytes (Figure 1E). Quantitative analysis of sarcomere spacing yielded very similar average values across conditions (Standard, 1.59 ± 0.17 μm; LGSF, 1.62 ± 0.10 μm; Adult CM, 1.70 ± 0.14 μm; Table 4), as expected, since well-aligned sarcomeres exhibit comparable periodicity regardless of culture conditions. The key difference therefore lies not in the absolute spacing, but in the extent and regularity of sarcomeric alignment across the cell volume. Representative α-actinin intensity profiles (Figure 1E) illustrate this difference, highlighting the globally uniform Z-band alignment achieved in LGSF cultures compared to the patchy organization typical of standard medium. All analyses were performed on day 15 of culture unless otherwise specified. This time point was selected based on previous evidence that functional neuro-cardiac junctions (NCJs) form at this stage [9], and was adopted here to ensure the synchronous maturation of both sympathetic neurons (SNs) and CMs.

#### 3.1.2. LGSF Medium Promotes Intercellular Structure Maturation

**Figure 2:**
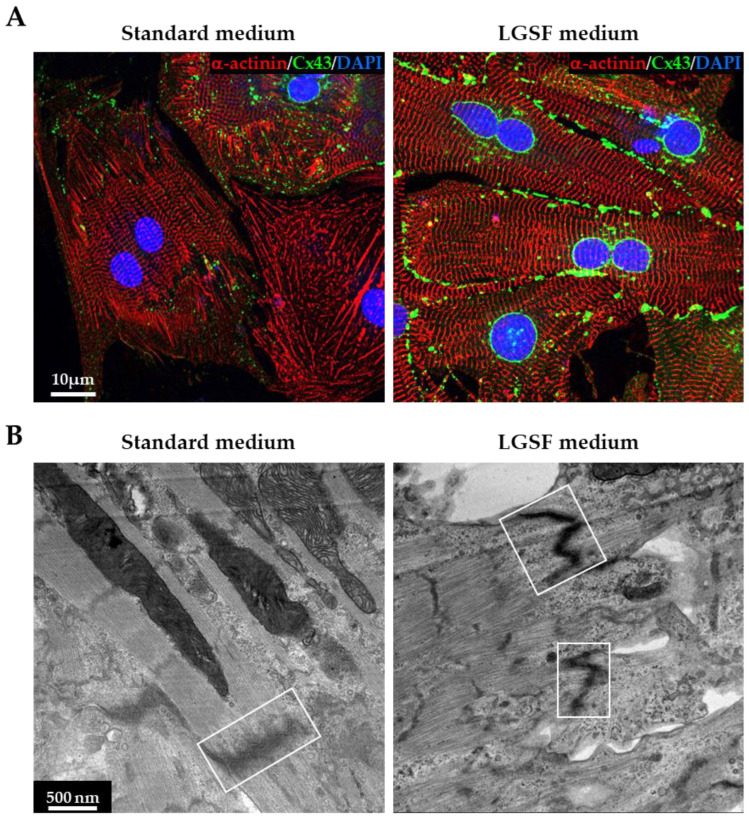
LGSF medium promotes maturation of intercellular structures. (**A**) Confocal images of neonatal cardiomyocytes (CMs) cultured (for 15 days) in standard medium (left) or low-glucose serum-free (LGSF) medium (right), stained for α-actinin (red) and Connexin-43 (Cx43, green). Nuclei were counterstained with 4′,6-diamidino-2-phenylindole (DAPI, blue). (**B**) Transmission electron microscopy of CMs under the same conditions. White boxes highlight desmosome-like electron-dense junctions, which are more prominent in LGSF-cultured cells.

The structural integrity and connectivity of CMs depend on cytoskeletal networks that interface with intercalated disks and the extracellular matrix. Connexin-43 (Cx43), the principal gap junction protein in the myocardium, plays a key role in the electrochemical coupling between adjacent CMs [28]. We evaluated Cx43 localization by immunofluorescence in neonatal CMs cultured under standard or LGSF conditions. In standard medium, Cx43 appeared as scattered puncta near the nucleus and diffusely within the cytoplasm. In contrast, LGSF-cultured CMs displayed stronger, polarized Cx43 staining at the cell periphery, particularly at intercellular contact sites, consistent with enhanced formation of functional gap junctions (Figure 2A). These observations were supported by transmission electron microscopy, which revealed more densely packed and structurally organized intercalated disks in CMs maintained under LGSF conditions (Figure 2B). Together, these findings indicate that LGSF medium promotes the maturation of intercellular structural components in neonatal CMs, supporting the development of morphologically and functionally competent junctional complexes.

#### 3.1.3. LGSF Medium Influences Intracellular Organelle Maturation and Distribution

**Figure 3:**
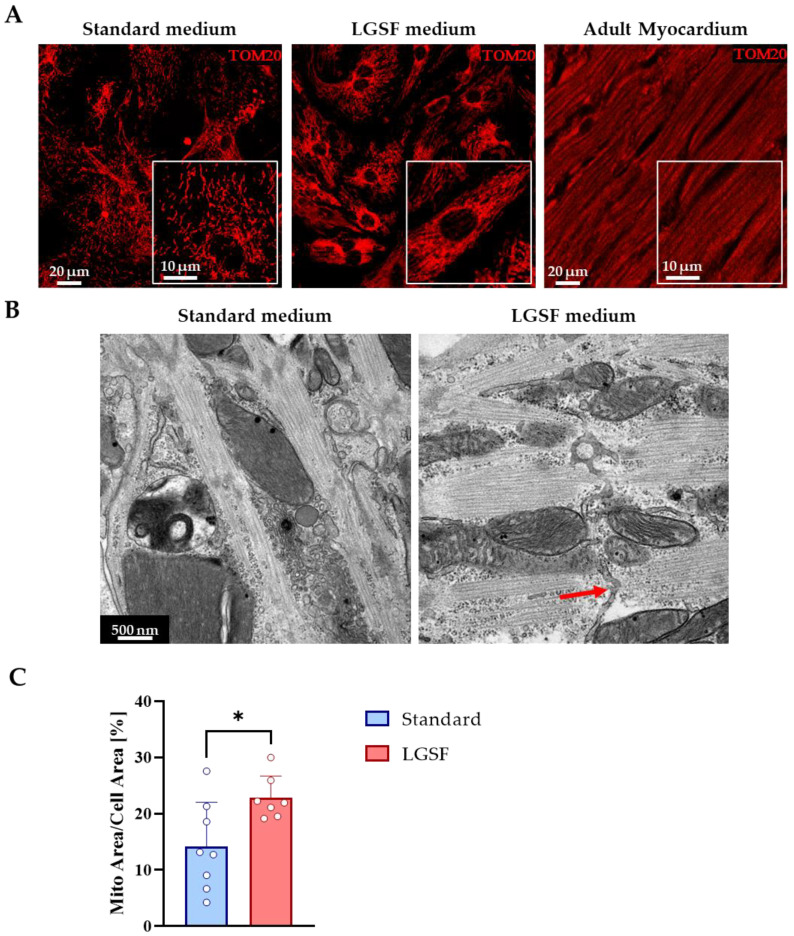
LGSF medium enhances mitochondrial distribution and ultrastructural maturation of the sarcoplasmic reticulum (SR). (**A**) Confocal images of neonatal cardiomyocytes (CM) cultured for 15 days in standard medium (left) or low-glucose serum-free (LGSF) medium (middle), compared to adult myocardium (right), stained for TOM20 (red) to visualize mitochondria. Insets show higher-magnification views. (**B**) Transmission electron micrographs under the same condition. Red arrow indicates sarcoplasmic reticulum-like tubular structures in LGSF-cultured cells. (**C**) Quantification of the mitochondrial area relative to cell size. Data represent pooled measurements from 20 cells per group, collected from *n* = 8 (Standard) and *n* = 7 (LGSF) coverslips across three independent cell cultures. Statistical analysis: unpaired two-tailed Student’s *t*-test; *, *p* ≤ 0.05.

**Figure 4:**
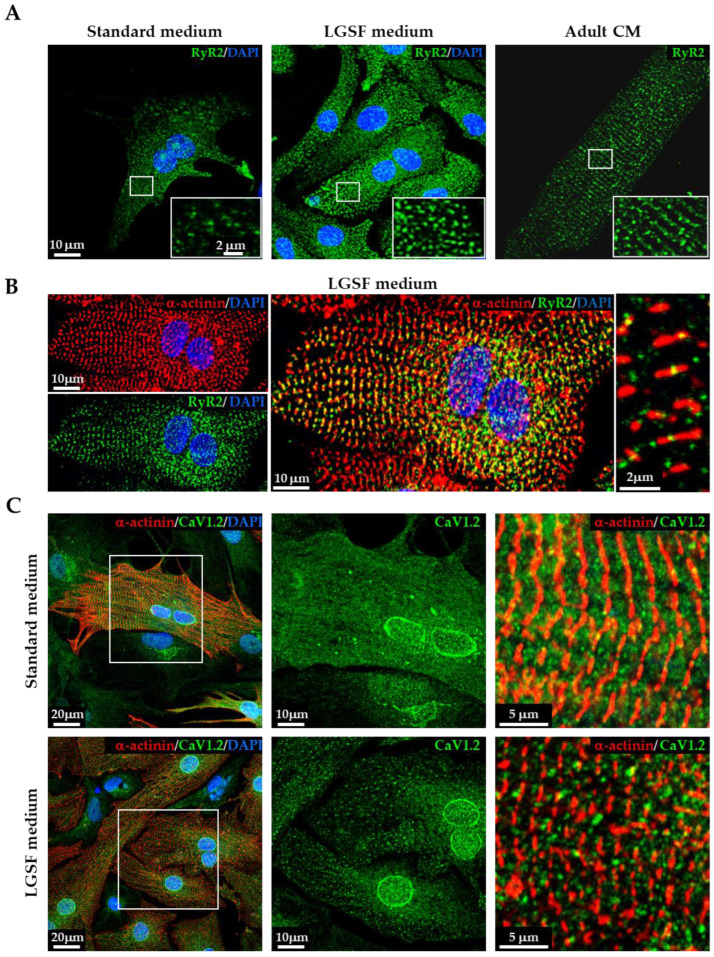
LGSF medium enhances sarcoplasmic reticulum and sarcolemmal maturation. (**A**) Confocal images of cardiomyocytes (CMs) cultured (for 15 days) in standard medium (left) or low-glucose serum-free (LGSF) medium (middle), compared to adult CMs (right), stained for RyR2 (green) and 4′,6-diamidino-2-phenylindole (DAPI, blue). (**B**) High-magnification confocal images of CMs cultured in LGSF medium stained for α-actinin (red) and RyR2 (green); merged image and magnified region highlight co-localization along sarcomeres. Nuclei were counterstained with DAPI (blue). (**C**) Confocal images of CMs stained for CaV1.2 (L-type Ca^2+^ channels, green) and α-actinin (red) in Standard (top) and LGSF (bottom) conditions. Nuclei were counterstained with DAPI (blue). Magnifications show increased co-localization in LGSF-cultured CMs. Data were collected from three independent culture experiments.

In adult CM, efficient excitation–contraction–metabolic coupling depends on the precise spatial organization of intracellular organelles, including mitochondria and the sarcoplasmic reticulum (SR). During postnatal development, mitochondria redistribute from a predominantly perinuclear pattern to an aligned arrangement along sarcomeres, while the SR matures into an intricate network with terminal cisternae positioned at Z-lines. Mitochondrial organization was assessed by TOM20 immunofluorescence. CMs cultured in standard medium exhibited clustered, perinuclear mitochondrial staining. In contrast, LGSF-cultured CMs showed a more adult-like mitochondrial pattern, with distribution across perinuclear, intermyofibrillar, and subsarcolemmal compartments (Figure 3A). This spatial reorganization was corroborated by transmission electron microscopy, which demonstrated a significantly greater mitochondrial area per cell under LGSF conditions (Standard: 14.1 ± 7.9% vs. LGSF: 22.8 ± 3.9%, *p* ≤ 0.05; Figure 3B,C, Table 4). To evaluate SR maturation, we analyzed the distribution of ryanodine receptor 2 (RyR2) by immunofluorescence. In CM maintained in LGSF medium, RyR2 clusters appeared more regularly aligned along sarcomeric Z-lines, with inter-cluster distances approaching those observed in adult CMs (Figure 4A,B). Similarly, L-type Ca^2^^+^ channels (LTCC) displayed increased fluorescence intensity and a more organized, striated pattern, consistent with enhanced membrane clustering and alignment with underlying RyR2 (Figure 4C). This spatial reorganization suggests improved functional coupling between LTCCs and RyR2 during excitation–contraction coupling (ECC). In line with these observations, protein levels of SERCA2A—the Ca^2^^+^ ATPase responsible for cytosolic Ca^2^^+^ reuptake into the SR—were significantly increased under LGSF conditions (Supplementary Figure S1B,C). Importantly, transmission electron microscopy not only confirmed enhanced mitochondrial content but also revealed a more mature SR architecture, featuring tubular extensions and terminal cisternae reminiscent of junctional SR domains in adult CMs (Figure 3B, red arrow). Collectively, these results indicate that the LGSF medium promotes the coordinated maturation of intracellular organelles and membrane specializations, including mitochondria, SR, and LTCC clusters, thereby establishing the subcellular architecture required for efficient ECC.

#### 3.1.4. LGSF Medium Improves Cytosolic and Mitochondrial Ca^2+^ Handling in Neonatal Cardiomyocytes

**Figure 5:**
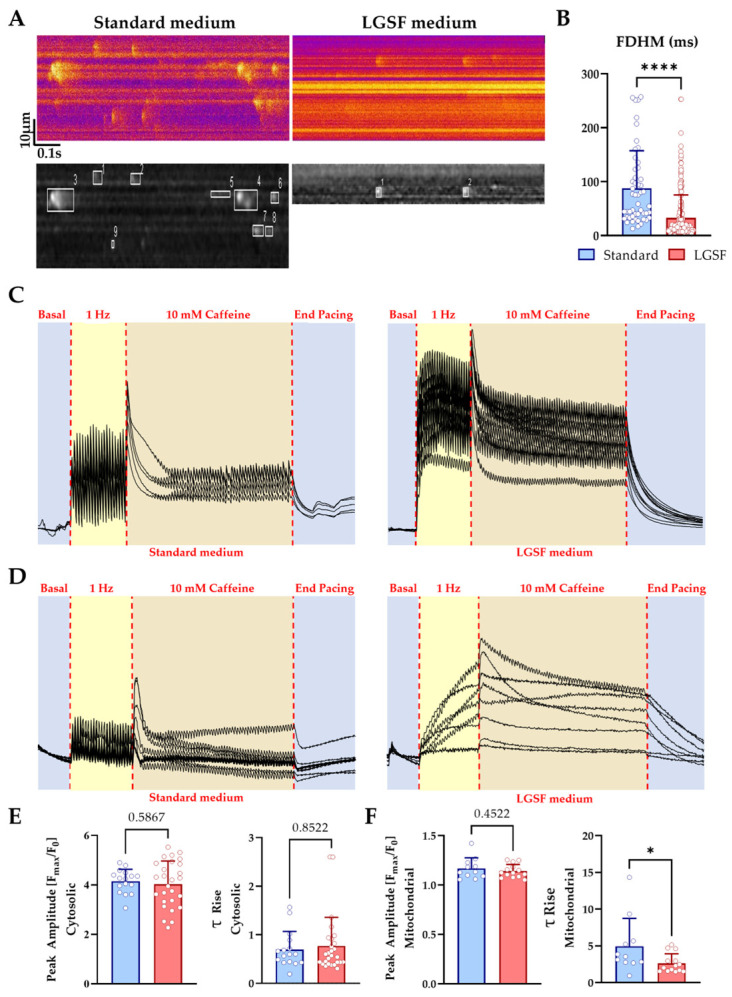
LGSF medium improves Ca^2+^ handling in neonatal cardiomyocytes (CMs). (**A**) Representative line-scan confocal images of Ca^2^^+^ sparks in CMs cultured for 15 days in standard (left) or LGSF (right) medium. Lower panels show Spark Master-processed images; boxes indicate detected Ca^2^^+^ events. (**B**) Quantification of full-duration at half-maximum (FDHM) of Ca^2^^+^ release events (Standard, *n* = 50 cells; LGSF, *n* = 190 cells). Mann–Whitney test; ****, *p* ≤ 0.0001. (**C**) Representative cytosolic Ca^2^^+^ transients during electrical pacing in CM cultured in standard (left) or LGSF (right) medium. (**D**) Representative mitochondrial Ca^2^^+^ traces during pacing in the same conditions. (**E**,**F**) Quantification of caffeine-induced Ca^2^^+^ peak amplitude and τ-rise in the cytosol (**E**) and mitochondria (**F**). Cytosolic Ca^2^^+^ quantification: standard, *n* = 16 cells; LGSF, *n* = 27 cells. Mitochondrial Ca^2^^+^ quantification: Standard, *n* = 11 cells; LGSF, *n* = 14 cells. Statistical analysis: unpaired two-tailed Student’s *t*-test (with Welch’s correction when required) or Mann–Whitney test. *, *p* ≤ 0.05. Data were collected from three independent culture experiments.

These architectural adaptations of the Ca^2^^+^ handling machinery prompted us to ask whether they translated into functional improvements in intracellular Ca^2^^+^ dynamics. Spontaneous and evoked Ca^2^^+^ signals were analyzed by real-time confocal imaging. In quiescent CM, standard medium produced large, prolonged macro-sparks and frequent Ca^2^^+^ waves, consistent with asynchronous and inefficient SR function. In contrast, LGSF-cultured CM exhibited smaller, spatially confined sparks with shorter duration and faster decay (FWHM, standard: 4.2 ± 3.1 vs. LGSF: 2.3 ± 2.0, in µm; FDHM, standard: 87.7 ± 69.8 vs. LGSF: 32.8 ± 42.6., in ms; *p* < 0.0001; Figure 5A,B, Table 4), indicative of more controlled Ca^2^^+^ release. Global cytosolic Ca^2^^+^ transients during 1 Hz pacing reached comparable amplitudes in both conditions (F/F_0_, standard: 3.40 ± 0.71 vs. LGSF: 3.15 ± 0.38; Figure 5C). Caffeine-induced SR release confirmed similar SR loading (Fmax/F_0_, standard: 4.03 ± 0.93 vs. LGSF 4.15 ± 0.48; Figure 5E) and no significant differences in release kinetics (τ-rise, standard: 0.77 ± 0.59 vs. LGSF: 0.70 ± 0.37). In addition to ECC, we assessed mitochondrial Ca^2^^+^ handling. LGSF-CM accumulated Ca^2^^+^ more rapidly during pacing (τ-rise, standard; 4.93 ± 3.80 vs. LGSF: 2.63 ± 1.29; Figure 5F), although maximal uptake after caffeine was similar (Fmax/F_0_, standard: 1.14 ± 0.07 vs. LGSF: 1.17 ± 0.11; Figure 5D,F). Together, these results show that LGSF medium enhances SR Ca^2^^+^ release fidelity and accelerates mitochondrial uptake in neonatal CM.

### 3.2. LGSF Medium Supports the Establishment of Functional Sympathetic Neuron–Cardiomyocyte Co-Cultures

**Figure 6:**
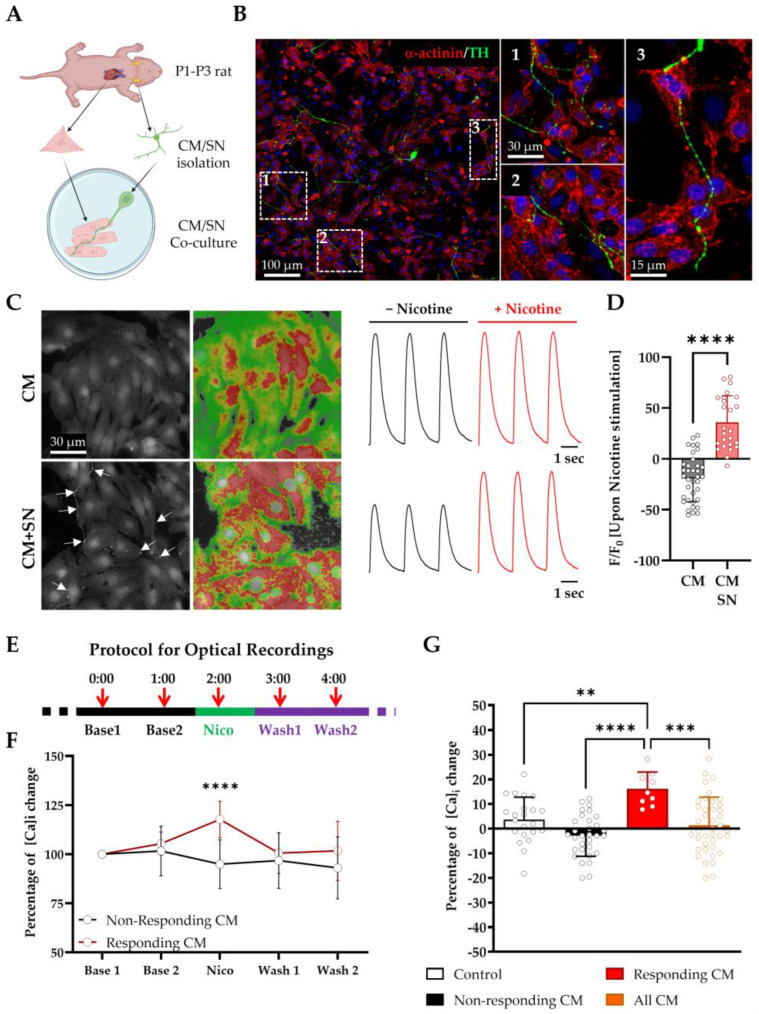
LGSF medium enables functional co-culture of neonatal cardiomyocytes (CMs) and sympathetic neurons (SNs). (**A**) Schematic of co-culture protocol using neonatal CMs and SNs from the same animals. (**B**) Immunofluorescence of 15 days of co-culture, showing α-actinin^+^ CMs (red) and tyrosine hydroxylase (TH)^+^ sympathetic neurons (SN, green). Right: higher magnification of boxed regions. (**C**) Fluo-4 Ca^2+^ imaging in mono- vs. co-cultures, before (black line) and after (red line) nicotine application. Pseudo-colored images show Ca^2+^ distribution; traces illustrate transient amplitude changes. White arrows identify SN neurites. (**D**) Quantification of Ca^2+^ transient amplitude fold increase upon nicotine superfusion (CM: *n* = 36; CMs plus SNs: *n* = 25). Unpaired *t*-test, ****, *p* ≤ 0.0001. (**E**) Schematic of Scanning Ion Conductance Microscopy (SICM)-guided local neuronal stimulation during Ca^2+^ imaging. (**F**) Amplitude increase in responsive vs. non-responsive CMs during SICM stimulation. Unpaired t-test, ****, *p* ≤ 0.0001 (non-responders: *n* = 31; responders: *n* = 9). (**G**) Grouped quantification of Ca^2+^ responses. One-way ANOVA with Tukey’s test (**, *p* ≤ 0.01; ***, *p* ≤ 0.001; ****, *p* ≤ 0.0001). Data were collected from three independent co-culture experiments.

Having shown that neonatal CMs acquire key hallmarks of maturation in LGSF medium, we next revisited our previously established co-culture system to investigate Ca^2^^+^-dependent neuro-cardiac interactions. In earlier studies, we employed this model to characterize the anatomical organization of the NCJ and to dissect the roles of neurotrophins and cAMP signaling in mediating bidirectional SN–CM communication [9,10,11]. Here, we build upon that framework by formally validating the culture conditions and expanding functional readouts to high-resolution Ca^2^^+^ imaging. In the intact heart, SNs form a specialized NCJ with CMs, allowing for beat-to-beat modulation of contractility via localized norepinephrine (NE) release. Recapitulating these interactions in vitro is technically challenging, due to the immature phenotype of cultured CMs and the fragility of SNs. To overcome these limitations, we optimized a protocol for the simultaneous isolation and co-culture of neonatal SNs and CMs from the same P1–P3 rats. CMs were seeded at 470 cells/mm^2^, and SNs added at a 1:50 SN:CM ratio previously shown to support efficient innervation [9,11] (Figure 6A). Co-cultures were established on laminin-coated surfaces and maintained in LGSF medium, which supports both cell types under serum-free, low-glucose conditions. After 72 h, tyrosine hydroxylase (TH)-positive SNs extended axonal projections, forming discrete varicosities—swellings consistent with neurotransmitter release sites—closely apposed to α-actinin-positive CM membranes (Figure 6B). Over time, this pattern evolved into a synaptic-like architecture resembling the in vivo “pearl-necklace” distribution of sympathetic varicosities. Based on previous studies demonstrating robust NCJ formation by day 15 [9,10,11], all functional analyses were performed at this time point. To assess neuro-cardiac functionality, co-cultures were loaded with Fluo-4 AM and paced at 0.5 Hz. Bath application of nicotine (1 µM), a nicotinic receptor agonist, triggered a significant increase in CMs’ Ca^2^^+^ transient amplitude (+36 ± 2% vs. baseline; *p* < 0.0001; Figure 6C,D). No effect was observed in CM-only cultures, confirming an SN-dependent response. To assess spatial specificity, nicotine was applied locally via SICM over individual SN soma, while Ca^2^^+^ signals were monitored in adjacent CMs (Supplementary Figure S2). Approximately 22% of CMs responded with enhanced Ca^2^^+^ transients, consistent with the proportion of CMs in direct contact with SN projections. Responses were reversible upon washout and colocalized post hoc with NCJ-like structures (Figure 6E–G).

These results demonstrate the following:

(i) LGSF medium supports SN survival, axonal growth, and the functional innervation of CMs;
(ii) SNs form anatomically and functionally defined NCJs;
(iii) SN activation modulates CM Ca^2^^+^ handling in a spatially restricted and reversible manner.

By formally validating the LGSF protocol and extending its application to functional Ca^2^^+^ imaging, we establish a robust and reproducible platform for high-resolution studies of neuro-cardiac communication.

### 3.3. β_1_- and β_2_- Adrenergic Receptors Differentially Contribute to Sympathetic Neuron-Evoked Ca^2^^+^ Responses in Cardiomyocytes

**Figure 7:**
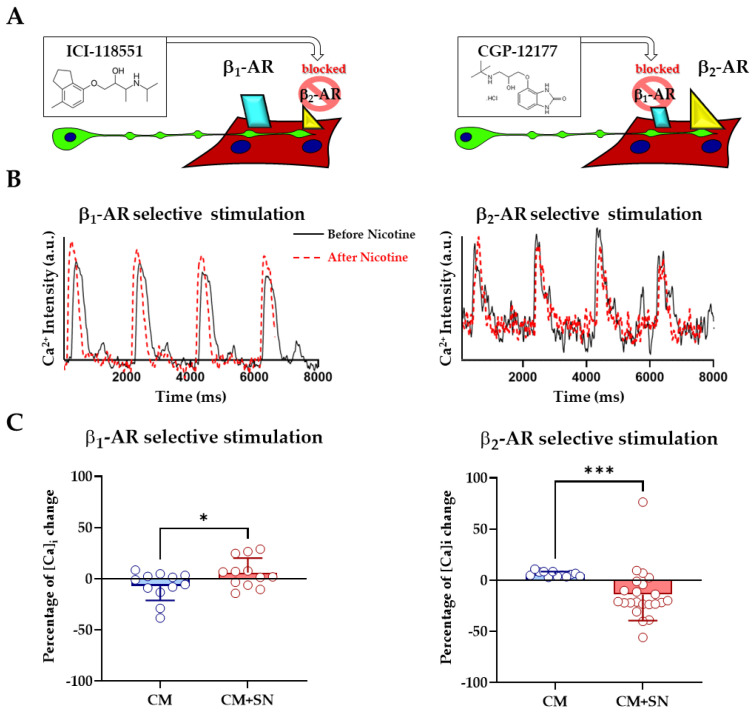
β_1_- and β_2_-adrenergic receptors differentially modulate neuron-evoked Ca^2+^ responses. (**A**) Experimental setup: 15 days of co-culture with ICI-118551 (β_2_-AR antagonist) or CGP-20712 (β_1_-AR antagonist), followed by nicotine stimulation. (**B**) Representative Ca^2+^ transients before (black line) and after (red dashed line) nicotine application under β_2_-AR blockade (left) or β_1_-AR blockade (right). (**C**) Quantification of fold increase in Ca^2+^ transient amplitude in mono- (CM) vs. co-cultures (CM + SN) under β_1_-AR (left) or β_2_-AR (right) stimulation. Mann–Whitney test (β_2_-AR) or unpaired t-test (β_1_-AR); *, *p* ≤ 0.05, ***, *p* ≤ 0.001. (β_1_-AR: CM, *n* = 12; CM plus SN, *n* = 12; β_2_-AR: CM, *n* = 12; CM plus SN, *n* = 22). Data were collected from three independent co-culture experiments.

Building on our previous work on β-adrenergic receptor (β-AR)-driven cAMP signaling in SN-CM co-cultures [9], we next investigated how β_1_- and β_2_- AR subtypes individually modulate CM Ca^2^^+^ dynamics in response to sympathetic activation. Both receptors are expressed in CM but exert distinct effects: β_1_-AR typically enhances contractility via the PKA-mediated phosphorylation of Ca^2^^+^-handling proteins, while β_2_-AR signaling can be more variable—sometimes even inhibitory—due to dual Gs/Gi-coupling and compartmentalized downstream pathways [29,30]. Our system exhibited elevated expression of both α- and β-AR transcripts (Supplementary Figure S3), consistent with a shift toward a more mature adrenergic phenotype. To dissect their respective contributions, we performed Ca^2^^+^ imaging in electrically paced SN–CM co-cultures following the selective pharmacological inhibition of each receptor subtype (Figure 7A). Sympathetic activation was induced by bath application of nicotine (1 µM), and CM Ca^2^^+^ transients were recorded in the presence of either ICI-118551 (50 nM, β_2_-AR antagonist) or CGP-20712 (300 nM, β_1_-AR antagonist). Selective β_1_-AR activation (i.e., β_2_-AR blockade) led to a modest but significant increase in Ca^2^^+^ transient amplitude in co-cultures compared to CM alone (Ca^2^^+^ change: CM only, –7 ± 4% vs. CM + SN, +6 ± 4%; *p* < 0.05). Conversely, β_2_-AR stimulation (β_1_-AR blocked) resulted in a decrease in Ca^2^^+^ transient amplitude (CM + SN, –14 ± 5%; *p* < 0.05; Figure 7B,C), in line with previously reported inhibitory β_2_-AR effects under high-stimulation conditions. Interestingly, the Ca^2^^+^ response observed during selective β_1_-AR stimulation (+6 ± 4%) was substantially lower than the full response observed without receptor blockade (+36 ± 5%; Figure 6D and Figure 7B,C), suggesting cooperative or synergistic interactions between β_1_- and β_2_-AR in mediating maximal sympathetic responsiveness. These findings demonstrate that our model can resolve receptor-specific signaling dynamics, offering a powerful platform to dissect the nuanced regulation of neuro-cardiac communication during CM maturation.

## 4. Discussion

Neonatal rodent cardiomyocytes (CMs) are a widely adopted in vitro model in cardiovascular research due to their ease of isolation, genetic manipulability, and long-term viability in culture. Importantly, their basic signaling pathways and transcriptional programs are preserved relative to adult CMs, making them particularly attractive for mechanistic studies [1,4]. However, a major limitation of this model lies in the immature phenotype of neonatal CMs, which differ from their adult counterparts in terms of morphology, cytoarchitecture, metabolic specialization, Ca^2^^+^ handling, and responsiveness to external stimuli. These limitations are particularly restrictive when studying excitation–contraction coupling (ECC), autonomic modulation, or intercellular communication in physiologically relevant contexts. Here, we addressed these limitations by developing a defined low-glucose, serum-free (LGSF) medium that enhances the structural and functional maturation of neonatal CMs, supporting their use in advanced applications such as sympathetic neuron (SN)-CM co-culture and subtype-specific β-adrenergic receptor (AR) signaling analyses. Compared to standard culture protocols, which rely on high-glucose DMEM and serum supplementation, our approach more closely mimics the postnatal in vivo environment, promoting coordinated maturation across cellular and subcellular compartments. Notably, robust structural and functional improvements were already evident on day 15 of culture, a time point we selected based on prior evidence showing that the neuro-cardiac junction (NCJ) matures at this stage [9]. This temporal alignment between SNs and CMs enhances their physiological relevance in co-culture systems. However, the benefits of the LGSF medium extend well beyond neuro-cardiac studies. By promoting the coordinated maturation of sarcomeres, mitochondria, and the sarcoplasmic reticulum (SR), this defined culture system provides a robust platform for investigating multiple aspects of cardiac cell biology—including ECC, β-AR responsiveness, hypertrophic signaling, and Ca^2+^ dynamics across cytosolic and organellar compartments. Importantly, the improved maturity of CMs under LGSF conditions enables the study of neuro-cardiac interactions in a setting that captures key features of both physiological and pathological relevance. Within these dimensions, the data consistently indicate that LGSF drives a phenotype that is more mature than conventional neonatal cultures, yet still distinct from fully adult CMs. For example, sarcomere alignment, mitochondrial development, and Ca^2^^+^ handling are markedly improved, but complete t-tubule formation and adult-like electrophysiological properties are not achieved. We therefore define this as an intermediate stage of maturation. These limitations reflect the fact that certain hallmark features of adult CM maturity—such as fully developed T-tubule networks—are absent or incomplete in neonatal cultures [31]. As such, this model is not only well-suited to dissecting fundamental mechanisms of cardiac development and function, but also for exploring disease-associated remodeling, altered autonomic regulation, and maladaptive CM–neuron communication. Therefore, LGSF medium represents a versatile and reproducible tool for mechanistic studies and early-stage translational research in cardiovascular physiology and pathophysiology.

### 4.1. LGSF Medium Promotes Structural and Functional Maturation of Neonatal Cardiomyocytes

Standard CM culture protocols typically include high-glucose DMEM (25 mM) supplemented with 10–15% serum, which supports cell viability but also maintains CMs in a fetal-like state and promotes fibroblast proliferation [32,33]. Serum contains undefined and batch-variable mixtures of growth factors, cytokines, and mitogens that interfere with CM maturation. Moreover, high glucose concentrations have been shown to repress differentiation and delay the metabolic switch toward oxidative phosphorylation, a hallmark of postnatal CM development [26,34,35]. To overcome these issues, we formulated a low-glucose (5.5 mM) MEM-based medium, removing serum after the initial plating phase and replacing it with defined supplements (insulin, transferrin, selenium) that support cell survival and differentiation [36,37]. Culturing on laminin-coated surfaces, rather than collagen or gelatin, further promoted CM adhesion and cytoskeletal development, as laminin plays a key role in CM maturation [1,38,39]. These changes led to robust improvements in CM morphology and organization. CMs cultured in LGSF medium adopted a more rod-like shape, increased in area, and exhibited greater sarcomeric alignment and Z-line regularity, closely resembling adult CMs. Connexin-43 (Cx43), the main gap junction protein, relocalized to intercellular contact sites, forming organized intercalated disk-like structures visible by both immunofluorescence and electron microscopy. Of note, we also observed nuclear envelope localization of Cx43, a pattern associated with transcriptional regulation during cardiac development [40]. At the subcellular level, TOM20 and electron microscopy analyses revealed a redistribution of mitochondria from a perinuclear cluster to intermyofibrillar and subsarcolemmal zones, matching the pattern of adult CMs [41,42,43]. Relative mitochondrial volume also increased, suggesting enhanced biogenesis. In parallel, the SR developed into a more organized structure, with RyR2 and L-type Ca^2^^+^ channel (LTCC) clusters aligning along sarcomeric Z-lines [44,45]. These features are consistent with the assembly of functional Ca^2^^+^ release units (CRU), the anatomical foundation of efficient ECC.

### 4.2. LGSF Conditions Improve Excitation–Contraction Coupling and Ca^2^^+^ Cycling

One of the most striking effects of the LGSF medium was observed in Ca^2^^+^ handling properties, a functional hallmark of CM maturity. In standard cultures, neonatal CMs predominantly depend on extracellular Ca^2^^+^ influx via LTCC and the sodium–Ca^2^^+^ exchanger (NCX) in reverse mode, due to the underdeveloped SR [1,46,47,48,49]. Conversely, adult CMs rely on Ca^2^^+^-induced Ca^2^^+^ release (CICR) from the SR, mediated by tightly coupled dyads formed by LTCC and RyR2 clusters [44,45,50].

In our system, LGSF-cultured CMs displayed the following:

(i) Higher spatial confinement of Ca^2^^+^ sparks;
(ii) Reduced spontaneous wave activity;
(iii) Improved mitochondrial Ca^2^^+^ uptake velocity.

These findings indicate a functional maturation of the CICR machinery, with better coupling between depolarization, SR Ca^2^^+^ release, and mitochondrial Ca^2^^+^ import. The improved ECC phenotype was also supported by the anatomical organization of CRU and their alignment with sarcomeric Z-bands, features rarely achieved under standard culture conditions.

### 4.3. A Functional In Vitro Model of Sympathetic Innervation

The ability to model autonomic modulation in vitro is critically dependent on both CM maturity and the survival/integration of SNs. Previous co-culture systems often used immature CMs or immortalized neurons, or lacked defined junctional structures. Our optimized system overcomes these limitations by combining mature neonatal CMs with primary SNs isolated from the same animals and co-cultured under LGSF conditions. Within 72 h, SNs extended TH-positive axons decorated with varicosities, the presumed sites of NE release, in close contact with CM membranes. This “pearl necklace” distribution mimics the in vivo architecture of the NCJ [8]. Upon global stimulation with nicotine, we observed a robust, neuron-specific increase in CM Ca^2^^+^ transients, absent in CM-only controls. To probe the spatial specificity of the response, we applied nicotine focally via SICM, targeting individual neuronal soma. Only ~22% of CMs responded, matching the fraction in direct contact with TH-positive projections, which we classified as innervated CMs according to established morphological criteria (see Methods; [9]). These results confirm that our system enables the formation of anatomically and functionally defined NCJs, where adrenergic modulation is local, reversible, and physiologically relevant. Notably, these findings build upon our earlier studies, where we demonstrated the existence of synapse-like NCJ structures and their role in modulating cAMP dynamics [9,10,11]. The present data extend that work by revealing that NCJs also mediate Ca^2^^+^-dependent responses with spatial precision, further supporting their functional analogy to synaptic transmission. While our primary aim was to establish that the defined LGSF medium enhances CM maturation and supports reproducible NCJ formation, we acknowledge that direct sympathetic contact can exert additional trophic effects. In Pianca et al. [10], we demonstrated that CMs in contact with SNs exhibit an increased cell size, consistent with adrenergic influence on growth and organization. In the present study, however, we did not systematically dissect the maturation parameters specifically attributable to neuron co-culture (e.g., membrane potential, sarcomere spacing, mitochondrial network). Importantly, acute nicotine stimulation was used here solely as a functional probe of junctional signaling and Ca^2^^+^ dynamics, rather than as a maturational paradigm. Prior evidence indicates that chronic adrenergic stimulation can promote structural maturation [10], and in parallel studies we are currently assessing whether prolonged exposure to a low number of neurotransmitters, such as NE or NPY, can further enhance CM maturation within the LGSF system. Our previous work [9] quantitatively demonstrated that NCJs in neonatal co-cultures are structurally stable (varicosity enlargement from 0.85 ± 0.23 µm^2^ at 5 days to 1.38 ± 0.10 µm^2^ at 15 days, plateauing thereafter), ultra-structurally tight (average cleft ~42 nm), and functionally restricted to directly contacted CMs, which display significantly higher β-AR/cAMP–PKA responses compared to non-innervated cells. In the present study, we did not repeat the full morphometric census, but we show that, under LGSF conditions, NCJs reform reproducibly and support the receptor-specific sympathetic modulation of Ca^2^^+^ handling, thus validating LGSF as a permissive and robust environment for neuro–cardiac interactions.

### 4.4. Receptor-Specific Dissection of β-Adrenergic Signaling

A key advantage of our co-culture system lies in its ability to resolve β-AR signaling at the subtype level in a physiologically relevant, synaptically controlled context. Neonatal CMs express both β_1_- and β_2_-AR [51], but their roles in modulating Ca^2^^+^ handling and contractility remain incompletely understood, especially during early postnatal maturation [52,53,54]. By applying selective antagonists during SN stimulation, we found that β_1_-AR activation increased the Ca^2^^+^ transient amplitude, consistent with classical PKA-mediated inotropic effects, whereas β_2_-AR activation reduced Ca^2^^+^ amplitude, in line with prior reports of inhibitory or biphasic actions in neonatal or stressed CM [29]. Notably, the inhibition of β_2_-AR blunted the β_1_-AR–mediated response, suggesting functional crosstalk, possibly via the β_2_-driven compartmentalization of β_1_ signaling. These data confirm the functional maturity and receptor responsiveness of CMs under LGSF conditions and demonstrate the utility of our system for dissecting neuro-modulatory pathways at single-cell and junctional resolutions. In parallel, we observed a significant upregulation of β_1_-, β_2_-, and α-AR transcripts over time, consistent with increased sympathetic input and progressive CM differentiation. However, the β_1_:β_2_ ratio remained lower than in adult CMs, suggesting an intermediate maturation state in which adrenergic signaling is functionally competent but not yet adult-like. This observation further supports the notion that, although LGSF significantly enhances neonatal CM properties, the cells retain an intermediate phenotype when compared to fully adult CMs. Overall, our co-culture reliably recapitulates hallmark features of adrenergic regulation, including NCJ-specific responses, subtype-selective signaling, and dynamic morpho-functional plasticity. The present work demonstrates that, under LGSF conditions, sympathetic inputs exert the receptor-specific modulation of Ca^2^^+^ handling via β_1_- and β_2_-AR. While we did not include additional downstream readouts, such as PKA substrate phosphorylation or β-AR target gene expression, cAMP/PKA signaling at NCJs has been previously documented [9]. Future studies building on the LGSF system may extend these findings by integrating molecular analyses to dissect the β-AR signaling cascade in greater detail.

### 4.5. Limitations and Outlook

While our system achieves significant structural and functional maturation, it remains a 2D monolayer and lacks the biomechanical complexity of in vivo or engineered 3D models. Despite clear improvements in morphology, cytoarchitecture, and Ca^2^^+^ handling, LGSF-cultured CMs retain an intermediate maturation state, as hallmarks of full adult phenotype—such as complete t-tubule networks, mature electrophysiological profiles, conduction velocity, and upstroke kinetics—are not yet achieved [31,55]. The 2D configuration ensures accessibility and reproducibility but imposes inherent constraints: Ca^2^^+^ cycling is less synchronized due to incomplete t-tubules, conduction is isotropic rather than anisotropic, and sympathetic junctions cannot fully replicate the three-dimensional topography and mechano-electrical environment of the intact heart. Looking forward, the defined LGSF medium could be adapted to advanced 3D platforms, including engineered heart tissues, scaffold-based cultures, or mechanically/electrically conditioned systems, thereby enhancing physiological relevance while retaining the advantages of defined conditions.

### 4.6. Comparison with Other Protocols

A comparison with currently available protocols underscores the competitive advantages of our approach. While several studies have aimed to improve the maturation of neonatal or pluripotent stem cell-derived CMs, most rely on complex, undefined media formulations, extended culture periods, or external conditioning [56,57,58,59]. For instance, Ronaldson-Bouchard et al. achieved advanced maturation of human iPSC-derived cardiac tissues using progressive electrical stimulation in engineered constructs [57]. Mills et al. developed cardiac organoids with metabolic arrest, but the long protocols and limited accessibility hinder direct neuro-cardiac interrogation [58]. Similarly, Feyen et al. proposed a fatty acid-based medium that improved iPSC-CM maturation but lacked control over neuron–CM integration [59]. In contrast, our LGSF protocol achieves the robust morphological, metabolic, and electrophysiological maturation of neonatal CMs by day 15, without external stimulation. Given its accessibility, reproducibility, and defined nature, our system may also serve as a modular platform for preclinical drug testing, the screening of neuro-modulatory compounds, or even educational purposes in neuro-cardiac physiology. Beyond improving the structural and functional maturation of neonatal CMs, the LGSF culture system may also serve as a versatile platform for translational applications. The reproducibility and defined conditions make it well-suited to disease modeling, for example, investigating mechanisms of hypertrophic signaling, arrhythmogenic remodeling, or ischemia-induced injury in a controlled environment. In addition, the system provides a robust framework for pharmacological testing, enabling the assessment of drug responses to CM maturation, Ca^2^^+^ handling, and neuro-cardiac interactions under serum-free and standardized conditions. Such applications have the potential to broaden the relevance of this platform for both basic and preclinical research.

## 5. Conclusions

In summary, we introduce a defined low-glucose serum-free culture system that achieves the following:

(i) Enhances the structural and functional maturation of neonatal cardiomyocytes (CMs) beyond what is achievable with standard protocols;
(ii) Supports the formation of a physiologically relevant neuro-cardiac junction (NCJ) with sympathetic neurons (SN);
(iii) Enables the dissection of receptor-specific β-AR signaling in a controlled, synaptic environment.

Structural and functional improvements are already evident at day 15, consistent with our prior demonstration that the NCJ matures at this stage [9]. This time point also corresponds to peak morphological, metabolic, and adrenergic maturation, making it a reliable window for functional assays and pharmacological testing. At the same time, CMs continue to mature over time, offering flexibility for extended studies of cardiac remodeling or disease modeling. By formally characterizing the defined medium and demonstrating Ca^2^^+^-dependent, receptor-specific sympathetic modulation with both spatial and functional resolution, we establish a robust and versatile in vitro model for investigating neuro-cardiac interactions, cardiac maturation, and drug responses under defined and physiologically relevant conditions.

## Figures and Tables

**Table 1:** Standard and LGSF media composition. All components are from Thermo Fisher Scientific, Waltham, MA, USA.

Component	Standard Medium (% *v*/*v*)		LGSF Medium (% *v*/*v*)	
	1st Day	2nd Day	1st Day	2nd Day
DMEM (25 mM HEPES)	67	75	–	–
M199	17.5	17	–	–
Horse Serum (HS)	10	5	–	–
Newborn Calf Serum (NCS)	5	0.5	–	–
MEM	–	–	88.8	98.8
Fetal Bovine Serum (FBS)	–	–	10	0
Non-Essential Amino Acids (NEAA)	–	–	0.1	0.1
ITS-X Supplement	–	–	0	0.1
L-Glutamine	1	1	–	–
Penicillin/Streptomycin	1	1	1	1

**Table 2:** Antibodies used in this study.

Antibody	Host	Application	Dilution	Supplier	RRID
α-Actinin	M	IF	1:200	SA	AB_2221571
		WB	1:1000		
Actin	Rb	WB	1:2500	SA	AB_476738
RyR2	Rb	IF	1:200	SA	AB_1856527
CaV1.2 (LTCC)	Rb	IF	1:100	Alomone	AB_2039771
Connexin-43	Rb	IF	1:200	SA	AB_2294609
TOM20	Rb	IF	1:200	SCB	AB_2207533
Thyrosine Hydroxylase	Rb	IF	1:200	SA	AB_390204
SERCA2A	M	WB	1:1000	Invitrogen	AB_325502
Anti-Rb-488	G	IF	1:200	JL	AB_2338052
Anti-M-488	G	IF	1:200	JL	AB_2338840
Anti-M-Cy3	G	IF	1:200	JL	AB_2338692
Anti-Rb-Cy3	G	IF	1:200	JL	AB_2338006
Anti-M-HRP	G	WB	1:5000	BioRad	AB_2921252
Anti-Rb-HRP	G	WB	1:5000	BioRad	AB_11125142

SA: Sigma Aldrich; SCB: Santa Cruz Biotechnologies; JL: Jackson Laboratories; M: mouse; Rb: rabbit; G: goat; IF: immunofluorescence; WB: Western blot; LTCC: L-type calcium channel; HRP: horseradish peroxidase.

**Table 3:** Oligos used in this study.

Gene (Rat)	Forward Sequence (5′-3′)	Reverse Sequence (5′-3′)
*Adra1a*	GGTTGCTTCGTCCTCTGCT	GAAATCCGGGAAGAAAGACC
*Adra1b*	CCCTTCTTCATCGCTCTCC	GGATTGAGGCAGCTGTTGA
*Adra1d*	TTCTTCTTCGTCCTGCCTCT	AGCGGGTTCACACAGCTATT
*Adrb1*	AGAGCAGAAGGCGCTCAAG	AGCCAGCAGAGCGTGAAC
*Adrb2*	TGCTATCACATCGCCCTTC	ACCACTCGGGCCTTATTCTT
*Gapdh*	CACCATCTTCCAGGAGCGAG	CCTTCTCCATGGTGGTGAAGAC

**Table 4:** Morphologic and functional maturation of CMs upon culture with LGSF medium (after 15 days of culture).

Parameter	Neonatal CM in Standard Medium	Neonatal CM in LGSF Medium	Adult CM	*p*-Value (Standard vs. LGSF)
Aspect Ratio	2.00 ± 0.85	2.48 ± 1.03	5.41 ± 1.49	≤0.01
Roundness	0.57 ± 0.18	0.47 ± 0.18	0.19 ± 0.05	≤0.01
Solidity	0.73 ± 0.11	0.79 ± 0.11	0.85 ± 0.06	≤0.01
Cell Area (μm^2^)	800.8 ± 325.1	1017.9 ± 311.5	2109.47 ± 664.3	≤0.001
Sarcomere distance	1.59 ± 0.17	1.62 ± 0.10	1.70 ± 0.14	>0.9999
Mitochondrial Content [%]	14.1 ± 7.9	22.8 ± 3.9	~30% [26]	≤0.05
Spark FDHM [ms]	87.7 ± 69.8	32.8 ± 42.6	~20–30 [27]	≤0.0001
Spark FWHM [μm]	4.2 ± 3.1	2.3 ± 2.0	~2 [27]	≤0.0001

## Data Availability

The raw data supporting the conclusions of this article will be made available by the authors on request.

## Supplementary Materials

The following supporting information can be downloaded at: https://www.mdpi.com/article/10.3390/cells14181434/s1, Figure S1: LGSF medium increases culture purity of neonatal rat ventricular cardiomyocytes; Figure S2: Scanning ion conductance microscopy (SICM) setup for local neuronal stimulation; Figure S3: LGSF medium modulates adrenergic receptor expression in neonatal cardiomyocytes.

## Abbreviations

AR—adrenergic receptor; CICR—Ca^2+^-induced Ca^2+^ release; CM(s)—cardiomyocyte(s); CRU—Ca^2+^ release unit; Cx43—connexin-43; DMEM—Dulbecco’s Modified Eagle Medium; DAPI—4′,6-diamidino-2-phenylindole; ECC—excitation–contraction coupling; hiPSC-CM—human-induced pluripotent stem cell-derived cardiomyocytes; LGSF—low-glucose serum-free (medium); LTCC—L-type Ca^2+^ channel; MEM—minimum essential medium; NCJ—neuro-cardiac junction; NE—norepinephrine; NCX—sodium–Ca^2+^ exchanger; RT—room temperature; RyR2—ryanodine receptor type 2; SICM—scanning ion conductance microscopy; SN—sympathetic neuron(s); SR—sarcoplasmic reticulum; TH—tyrosine hydroxylase.
