# Supplementary material for: Defined Composition of Culture Media Promotes Rodent Neonatal Cardiomyocyte Maturation and Enables Functional Neuro-Cardiac Co-Culture
Source: Cells. 2025 Sep 13;14(18):1434. doi: 10.3390/cells14181434 (PMC12468161; doi:10.3390/cells14181434)
Supplement: Supplementary file 1 [file cells-14-01434-s001.zip › cells-3847260-supplementary.pdf]

cells

Article

Defined composition of culture media promotes rodent neonatal cardiomyocyte maturation and enables functional neuro-cardiac co-culture

Giulia Borile [1,2,*], Lolita Dokshokova [1,2,3,*], Nicola Moro [1,2,*], Antonio Campo [1,2], Valentina Prando [1,2], Jose L. Sanchez Alonso-Mardones [3], Julia Gorelik [3], Giuseppe Faggian [4], Marco Mongillo [1,2,§], Tania Zaglia [1,2,§]

[1] Department of Biomedical Sciences, University of Padova, Padova, Italy;
[2] Veneto Institute of Molecular Medicine, Padova, Italy;
[3] National Heart and Lung Institute, London, UK;
[4] Division of Cardiac Surgery, University of Verona, Verona, Italy.

* equal contribution
§ Correspondence:
marco.mongillo@unipd.it (M.M.), office number: +390497923229; fax number. +390497923250;
tania.zaglia@unipd.it (T.Z.), office number: +390497923294; fax number: +390497923250

Supplementary information

-Supplementary Figure Legends 1-3

Academic Editor: Firstname Last-name

Received: date
Revised: date
Accepted: date
Published: date

Citation: To be added by editorial staff during production.

Copyright: © 2025 by the authors. Submitted for possible open access publication under the terms and conditions of the Creative Commons Attribution (CC BY) license (https://creativecommons.org/licenses/by/4.0/).

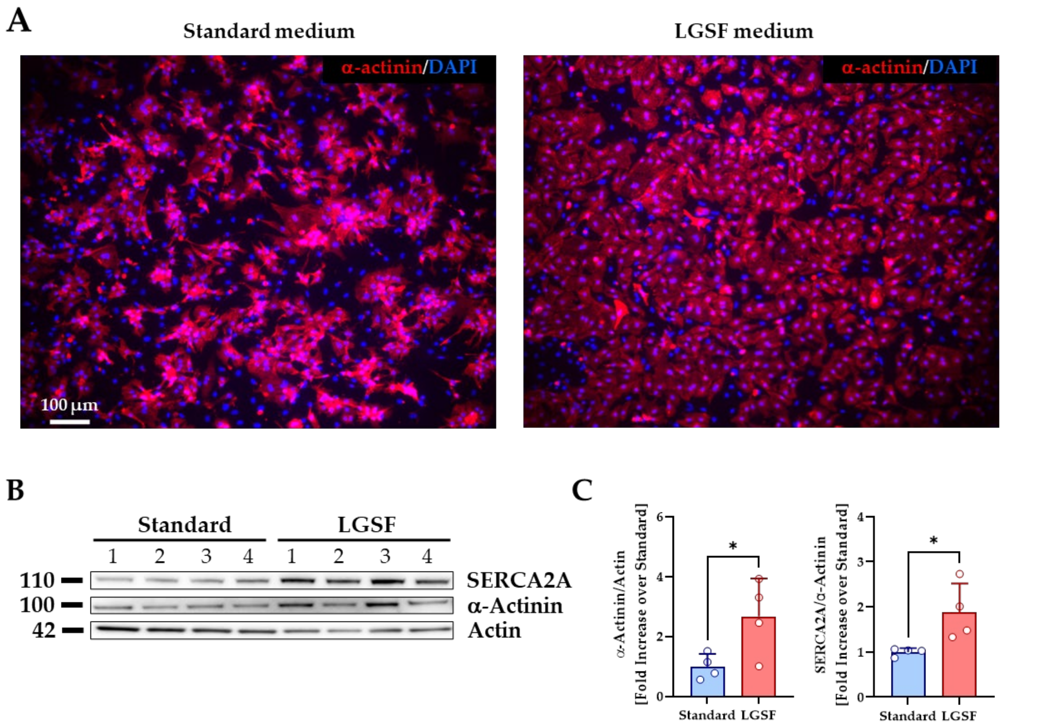

Supplementary Figure S1. LGSF medium increases culture purity of neonatal rat ventricular cardiomyocytes.

(A) Immunofluorescence staining of CM cultured in Standard (left) or Low-Glucose Serum-Free (LGSF) medium (right), showing α-actinin$^+$ CM (red) and 4',6-diamidino-2-phenylindole (DAPI) -stained nuclei (blue). Reduced fibroblast contamination is observed under LGSF conditions. **(B)** Western blot analysis of α-actinin and SERCA2A expression in protein lysates from CM cultured in Standard or LGSF medium. Actin was used as a loading control. **(C)** Densitometric quantification of α-actinin expression relative to Actin, and SERCA2A expression relative to α-actinin. Statistical analysis: unpaired t-test; *, $p \le 0.05$; $n = 4$ independent samples per group.

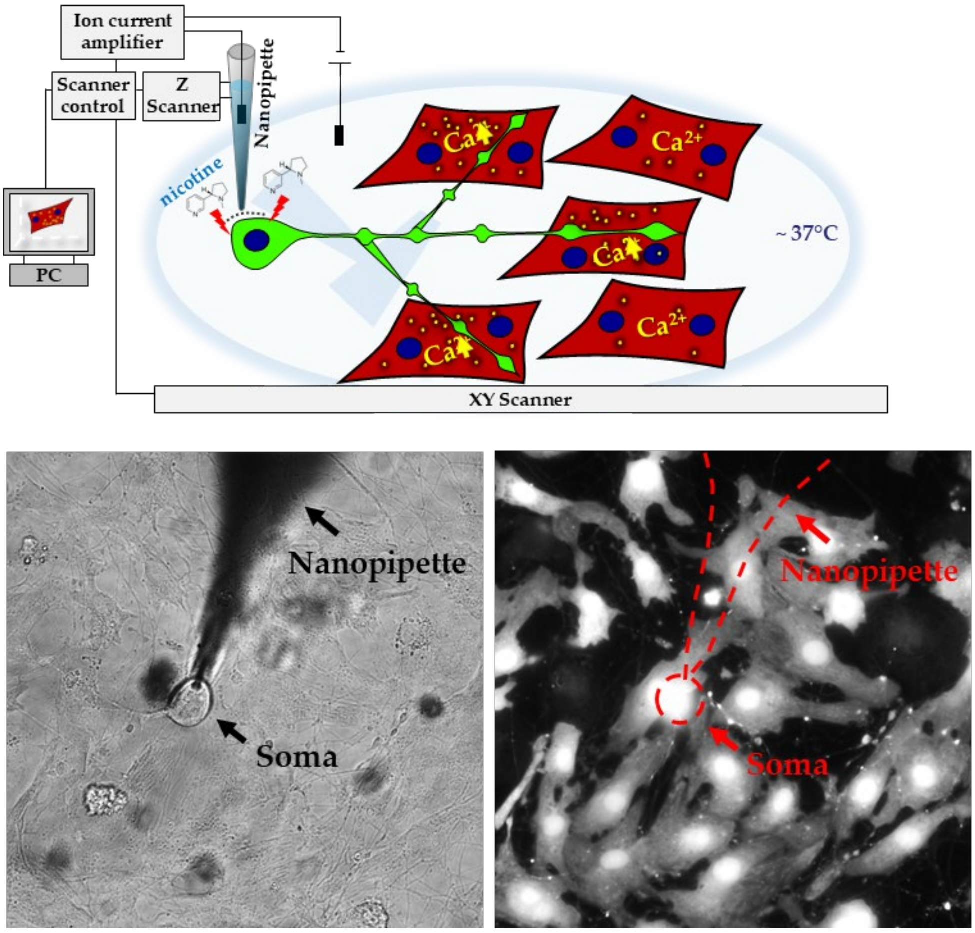

Supplementary Figure S2. Scanning Ion Conductance Microscopy (SICM) setup for local neuronal stimulation.

Schematic overview of the SICM-based stimulation protocol. After identifying the neuronal soma, a glass nanopipette (30 MΩ) is positioned above the cell. Nicotine is locally delivered via combined pressure and voltage pulses, while Ca^{2+} transients are simultaneously recorded in Fluo-4–loaded CM paced at 0.5 Hz within the same field of view.

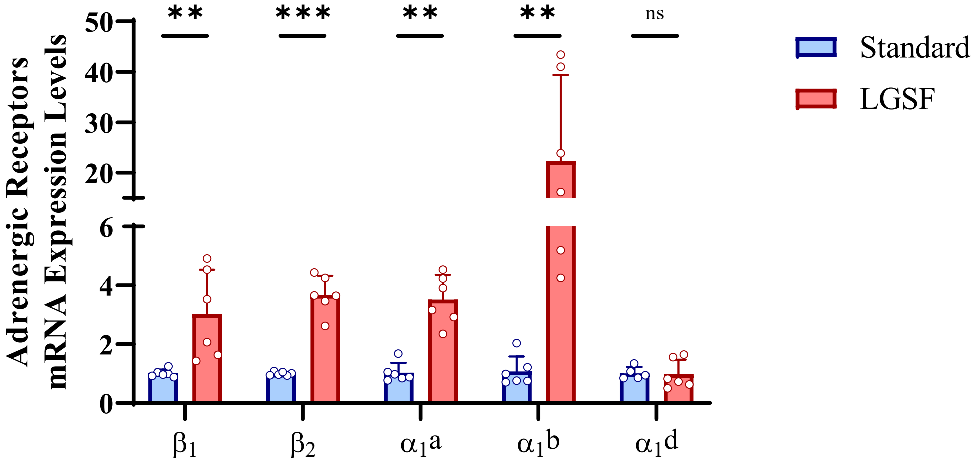

Supplementary Figure S3. LGSF medium modulates β-adrenergic receptor expression in neonatal cardiomyocytes.

RT-qPCR analysis of adrenergic receptor mRNA levels in CM cultured under Standard

(blue) or LGSF (red) conditions. Data are expressed as mean ± SD from n = 6 independent samples per group. Statistical analysis: unpaired t-test (with Welch's correction when required) or Mann–Whitney test; **, $p \leq 0.01$; ***, $p \leq 0.001$.
